# Genetic analysis of pregnancy loss and fetal structural anomalies by whole exome sequencing

**DOI:** 10.1186/s13023-024-03340-5

**Published:** 2024-09-09

**Authors:** Jingjing Xiang, Yang Ding, Hui Tang, Wei Zhang, Jun Mao, Quanze He, Qin Zhang, Ting Wang

**Affiliations:** The Affiliated Suzhou Hospital of Nanjing Medical University, Suzhou Municipal Hospital, Gusu School, Nanjing Medical University, Suzhou, 215000 Jiangsu China

**Keywords:** Pregnancy loss, Fetal structural anomalies, Whole exome sequencing, CNV, Variants

## Abstract

**Background:**

Whole exome sequencing (WES) has been recommended to investigate the genetic cause of fetal structural anomalies. In this retrospective study, we aimed to evaluate the diagnostic yield of WES in our cohort of families with pregnancy loss or termination of pregnancy due to structural anomalies.

**Methods:**

As aneuploidy, triploidy and copy number variations (CNVs) could be detected by exome-based CNV analysis, only WES is performed in this study. And the results of 375 cases assessed by WES were analyzed.

**Results:**

The overall detection rate was 32.3% (121/375), including aneuploidy and triploidy (7.5%, 28/375), CNVs (5.1%, 19/375) and single-nucleotide variants (SNVs) /insertions or deletions (Indels) (19.7%, 74/375). Among these, the diagnostic yield for likely pathogenic (LP) or pathogenic (P) CNVs is 4.8% (18/375), and the diagnostic yield for LP or P SNVs/Indels is 15.2% (57/375). And an additional 4.8% (18/375) of cases had CNVs or SNVs/Indels classified as variants of uncertain significance (VUS) with potential clinical significance.

**Conclusions:**

Our findings expand the known mutation spectrum of genetic variants related to fetal abnormalities, increase our understanding of prenatal phenotypes, and enable more accurate counseling of recurrence risk for future pregnancies.

**Supplementary Information:**

The online version contains supplementary material available at 10.1186/s13023-024-03340-5.

## Background

Pregnancy loss is defined as the spontaneous demise of a pregnancy, including non-visualized pregnancy loss, ectopic pregnancy, miscarriage and stillbirth [1]. Fetal structural abnormalities can be detected by prenatal ultrasound in 3–5% of pregnancies [2, 3]. When pregnancy loss or fetal structural anomalies occurred, conventional methods such as karyotype analysis, as well as chromosomal microarray analysis (CMA) were applied to determine the underlying genetic causes. Karyotype analysis can detect chromosomal aneuploidies and polyploidies, which may lead to miscarriage [4], while CMA can increase the detection rate by detecting clinically significant submicroscopic copy number variations (CNVs) in fetuses with abnormal sonographic findings or stillbirths [5, 6]. CMA is recommended as a first-tier approach for detection of CNVs in fetuses with structural anomalies in prenatal diagnosis [7, 8]. When standard CMA and karyotype analysis have failed to yield a definitive diagnosis, it is recommended that whole exome sequencing (WES) may be considered for a fetus with ultrasonic anomalies [9]. WES appears to be a promising tool to detect single-nucleotide variants (SNVs), small insertions or deletions (Indels) as well as CNVs in the coding regions of the human genome [10].

In this study, we retrospectively investigated the utility of WES as the initial testing strategy in the genetic diagnosis of fetuses from 375 families who had experienced pregnancy loss or termination of pregnancy due to fetal structural anomalies. The aim of the study was to identify the underlying genetic etiology, and estimate the risk of recurrence for subsequent pregnancies. Furthermore, follow-up investigations such as Sanger sequencing, quantitative PCR (qPCR) and RNA analysis were applied to confirm the variants or CNVs identified by WES and the effects of specific splicing variants respectively.

## Methods

### Study design and participants

This was a retrospective study of 375 families who had experienced pregnancy loss or pregnancy termination for fetal abnormality referred to our center for reproduction and genetics, the affiliated Suzhou hospital of Nanjing medical university, Suzhou, Jiangsu, China between November 2019 and January 2024, which was approved by the institutional ethics committee of the Affiliated Suzhou Hospital of Nanjing Medical University. Written informed consent was obtained from the parents of the fetuses. The inclusion criteria were as follows: (1) cases of miscarriage occurring before the 20th week of pregnancy; (2) cases of stillbirth occurring at or after 20 weeks of pregnancy; (3) cases underwent terminations of pregnancy due to isolated or multiple fetal structural anomalies observed by prenatal ultrasonography. The characteristics of each fetus including gestation age, maternal age, classification and major affected organ/system(s) were listed in Supplementary Tables 1, 2, 3 and 4.

### Whole exome sequencing and data analysis

Genomic DNA was extracted from the fetal tissues (*n* = 371) or chorionic villi (*n* = 12) and peripheral blood of their parents respectively. Whole exome sequencing (WES) was performed using the IDT xGen™ Exome Hybridization Panel v2 (Integrated DNA Technologies, USA) and Illumina NovaSeq 6000 platform (Illumina, USA). The sequencing reads were mapped to the human reference genome (hg19/GRCh37) by the Sentieon software package (https://www.sentieon.com/). The average sequencing depth on target bases should be over 100X, with over 96% of target bases being covered at least 20X. The SNVs and Indels were called by GATK (https://software.broadinstitute.org/gatk/). The variants were searched in the dbSNP (https://www.ncbi.nlm.nih.gov/snp/), 1000 Genomes Project database (https://www.internationalgenome.org/) and the Genome Aggregation Database (gnomAD) (http://gnomad.broadinstitute.org/). The pathogenicity of missense variants was predicted by REVEL (https://sites.google.com/site/revelgenomics/about) and CADD (https://cadd.gs.washington.edu/snv). SpliceAI (https://spliceailookup.broadinstitute.org/) was used to predict the splicing impact of variants. Multiple databases were also searched, such as Online Mendelian Inheritance in Man (OMIM, http://www.omim.org), Human Gene Mutation Database (HGMD, http://www.hgmd.org), ClinVar (http://www.ncbi.nlm.nih.gov/clinvar) and PubMed (http://www.ncbi.nlm.nih.gov/pubmed). The variants were classified according to the standards and guidelines for the interpretation of sequence variants released by the American College of Medical Genetics and Genomics (ACMG) and the Association for Molecular Pathology [11]. In addition, an internal coverage-based tool called CNVexon (Fulgent Genetics) was used to detect CNVs from WES data [12]. The identified CNVs were interpreted according to the technical standards for the interpretation and reporting of constitutional copy-number variants recommended by the ACMG and the Clinical Genome Resource (ClinGen) [13].

### Sanger sequencing

To verify the WES results, the identified candidate variants were amplified by polymerase chain reaction (PCR) using specific primer sets and genomic DNA from the fetuses and their parents respectively and sequenced in two orientations using an ABI 3500 Genetic Analyzer (Applied Biosystems, USA).

### RNA analysis

To analyze the splicing effects of variants, total RNA was extracted from fetal tissues using RNAprep Pure Tissue Kit (Tiangen, China) and reverse transcribed into complementary DNA (cDNA) by HiScript III 1st Strand cDNA Synthesis Kit (+ gDNA wiper) (Vazyme, China). PCR-amplified cDNA were analyzed by electrophoresis and Sanger sequencing to assess the effect of the variant on splicing.

### Quantitative PCR

Small deletions or duplications were confirmed by quantitative PCR (qPCR). Several exons of the genes within the CNV were selected and specific primers were designed to amplify these exons. qPCR were performed using SYBR Green PCR Master Mix (Applied Biosystems, USA) on an ABI 7500 system (Applied Biosystems, USA). Three replicates were performed for each sample, and the relative copy number was calculated by comparative 2-ΔΔCT method using *ALB* gene as the internal control. 2-ΔΔCT < 0.1 is considered to be a homozygous deletion, 0.3 < 2-ΔΔCT < 0.7 is considered to be a heterozygous deletion, 0.7 < 2-ΔΔCT < 1.3 is considered to be normal, and 2-ΔΔCT > 1.3 is considered to be a duplication.

## Results

### Cohort characteristics

**Fig. 1:**
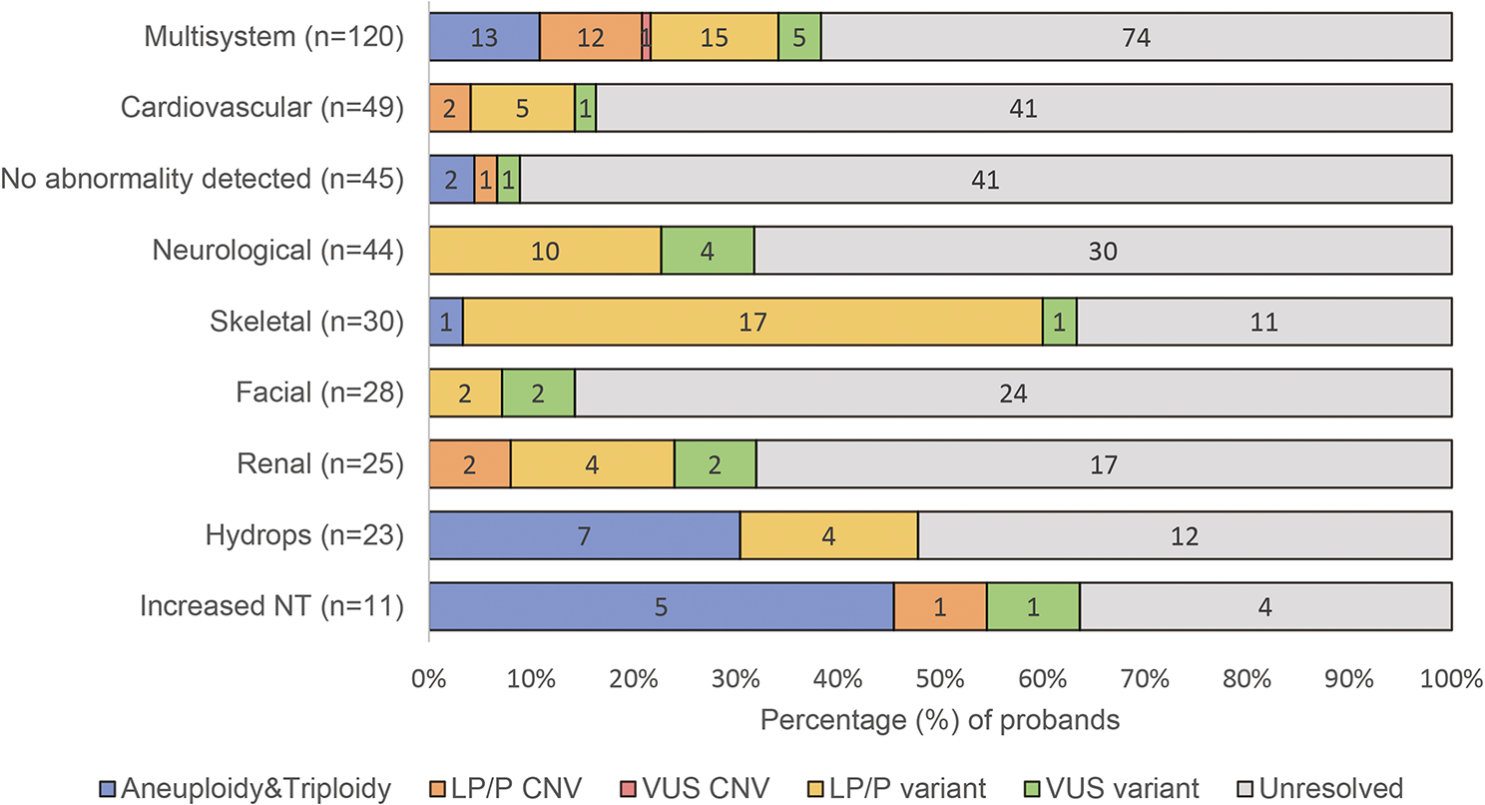
Distribution of cases and diagnostic rates per major organ/system(s) affected. Fetuses with ultrasonic abnormalities in ≥ 2 organ systems were defined as having multisystem anomalies. NT, nuchal translucency; Increased NT: NT > 3.0 mm; CNV, copy number variation; LP, likely pathogenic; P, pathogenic; VUS, variant of uncertain significance

This retrospective cohort includes fetuses of 375 families undergoing WES analysis. The median maternal age was 29 years (range 20–42), and the median gestational age was 23 weeks (range 8–38) (Supplementary Tables 1, 2, 3 and 4). The largest proportion of cases (83.5%, 313/375) were terminations of pregnancy due to structural anomalies, and the remaining were 33 cases of miscarriage (8.8%, 33/375) and 29 cases of stillbirth (7.7%, 29/375). Trio WES was performed for the majority of cases (76.3%, 286/375), and the remaining cases underwent singleton WES (21.3%, 80/375) or quad WES (2.4%, 9/375) (Table 1). According to the associated organ/ system of the abnormalities detected by ultrasound, the fetuses of 375 families were categorized into 9 phenotypic groups, including multisystem (≥ 2 organ systems, 32.0%, 120/375), cardiovascular (13.1%, 49/375), no abnormality detected (12.0%, 45/375), neurological (11.7%, 44/375), skeletal (8.0%, 30/375), facial (7.5%, 28/375), renal (6.7%, 25/375), hydrops (6.1%, 23/375) and increased nuchal translucency (NT) (2.9%, 11/375) (Fig. 1).

**Table 1:** Overview of the cohort characteristics and diagnostic yield

	Total number	%	Aneuploidy & Triploidy	%	LP/P CNV	%	VUS CNV	%	LP/P variant	%	VUS variant	%	Unresolved	%
**Total cases**	**375**		**28**	**7.5%**	**18**	**4.8%**	**1**	**0.3%**	**57**	**15.2%**	**17**	**4.5%**	**254**	**67.7%**
**Classification**														
Termination	313	83.5%	22	7.0%	17	5.4%	1	0.3%	56	17.9%	15	4.8%	202	64.5%
Miscarriage	33	8.8%	6	18.2%	1	3.0%	0	0.0%	0	0.0%	1	3.0%	25	75.8%
Stillbirth	29	7.7%	0	0.0%	0	0.0%	0	0.0%	1	3.4%	1	3.4%	27	93.1%
**Testing**														
Trio	286	76.3%	19	6.6%	14	4.9%	0	0.0%	48	16.8%	9	3.1%	196	68.5%
Quad	9	2.4%	0	0.0%	0	0.0%	0	0.0%	4	44.4%	1	11.1%	4	44.4%
Singleton	80	21.3%	9	11.3%	4	5.0%	1	1.3%	5	6.3%	7	8.8%	54	67.5%

CNV, copy number variation; LP, likely pathogenic; P, pathogenic; VUS, variant of uncertain significance. For 6 cases with more than one CNV, 1 case with 2 CNVs including a pathogenic CNV and a VUS CNV was listed in the group of LP/P CNV, and the rest 5 cases including 4 cases with 2 P CNVs and 1 case with a P CNV and a LP CNV were listed in the group of LP/P CNV. And 6 cases with compound heterozygous variants of a LP/P variant and a VUS variant were listed in the group of VUS variant.

### WES results

**Fig. 2:**
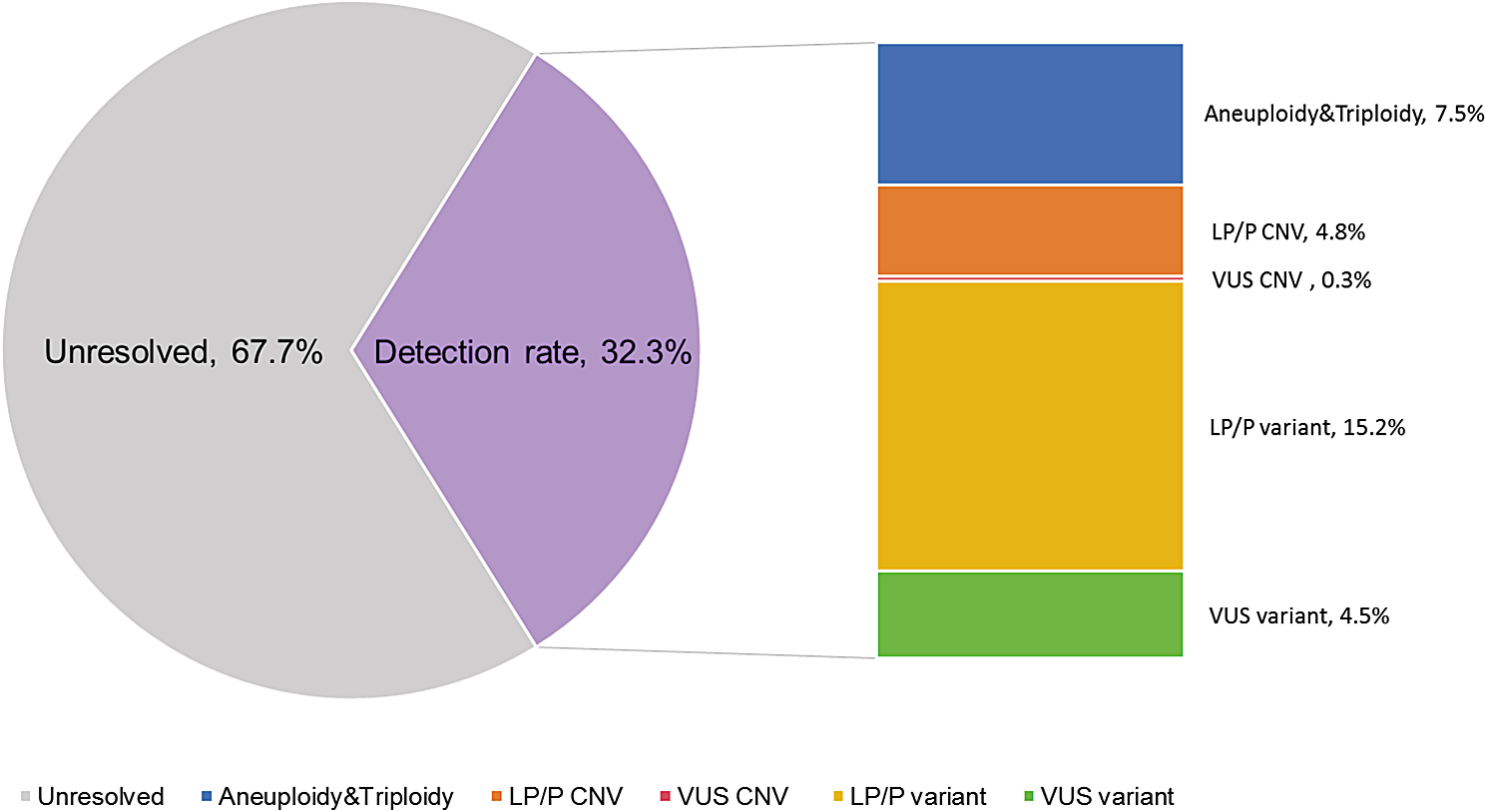
The overall detection rate of the cohort. CNV, copy number variation; LP, likely pathogenic; P, pathogenic; VUS, variant of uncertain significance

Among the fetuses of 375 families included, analysis by WES yielded an overall detection rate of 32.3% (121/375), of which aneuploidy and triploidy accounted for 7.5% (28/375), CNVs accounted for 5.1% (19/375) and SNVs/Indels accounted for 19.7% (74/375) (Fig. 2; Table 1). Among the three subgroups in Table 1, the diagnostic yield of aneuploidy and triploidy was the highest in the subgroup of miscarriage (18.2%, 6/33), and the detection rates of CNVs (5.8%, 18/313) and SNVs/Indels (22.7%, 71/313) were the highest in the subgroup of terminated pregnancies respectively. And the subgroup of stillbirth was associated with the lowest frequency of detection rate (6.9%, 2/29). The detection rate of Quad-WES was the highest (55.6%, 5/9), and the detection rates of Trio-WES (31.5%, 90/286) and single WES (32.5%, 26/80) were similar (Table 1). The yield of WES analysis varied considerably between the phenotypic groups. The greatest proportions of genetic variants were observed in fetuses with increased NT (63.6%, 7/11) and skeletal anomalies (63.3%, 19/30), followed by 47.8% in hydrops (11/23), 38.3% in multisystem (46/120), 32% in renal (8/25), 31.8% in neurological (14/44), 16.3% in cardiovascular (8/49) and 14.3% in facial (4/28). The lowest detection rate was found in cases of miscarriage or stillbirth without any congenital abnormalities (8.9%, 4/45) (Fig. 1).

28 cases of aneuploidy and triploidy include 9 cases of Trisomy 21, 8 cases of Turner Syndrome, 7 cases of Trisomy 18, 2 cases of Trisomy 13, 1 case of Trisomy 16, and 1 case of 69, XXY (Supplementary Table 1). Exome-based CNV calling identified 25 CNVs in 19 cases, and 23 CNVs were classified as pathogenic (P) or likely pathogenic (LP), 2 CNVs were classified as variants of uncertain significance (VUS) (Supplementary Table 2).

**Fig. 3:**
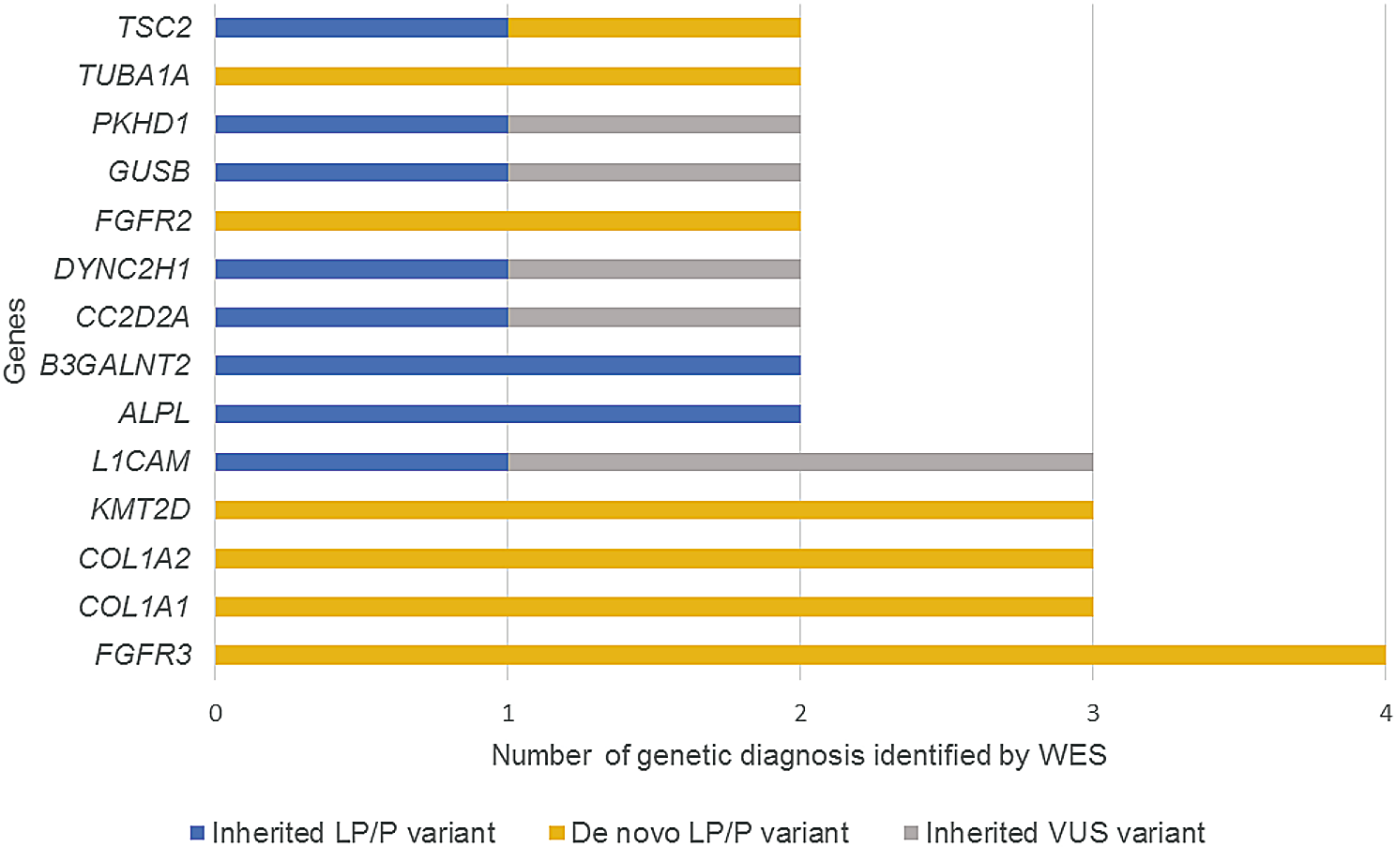
Genes with variants in more than one case. LP, likely pathogenic; P, pathogenic; VUS, variant of uncertain significance

Disease-causing SNVs/Indels of 54 genes were detected in 74 families, including 30 pathogenic (P) variants, 51 likely pathogenic (LP) variants and 18 variants of uncertain significance (VUS). Of these, 53 were novel variants not reported in public databases previously (Supplementary Table 3). De novo mutations were identified in 36 cases with autosomal dominant (33) or X-linked (3) inheritance, while the remaining 38 cases had inherited mutations, including 26 cases with autosomal recessive inheritance, 8 cases with autosomal dominant inheritance and 4 cases with X-linked inheritance (Table 2, Supplementary Table 3). The most prevalent disease was thanatophoric dysplasia caused by variants of *FGFR3* detected in 4 cases. Variants in 4 genes *L1CAM*, *KMT2D*, *COL1A2* and *COL1A1* were identified in 3 cases respectively, and variants in 9 genes were detected in 2 cases respectively (Fig. 3).

**Table 2:** Inheritance pattern of genes in 74 diagnosed cases

Mode of inheritance	*n*	%
**Autosomal dominant**	**41**	**55.4%**
De novo	33	44.6%
Inherited	8	10.8%
**Autosomal recessive**	**26**	**35.1%**
Compound heterozygous	25	33.8%
Homozygous	1	1.4%
**X-linked (dominant**,** recessive**,** both)**	**7**	**9.5%**
De novo	3	4.1%
Inherited	4	5.4%

### Case examples

#### Case 8

**Fig. 4:**
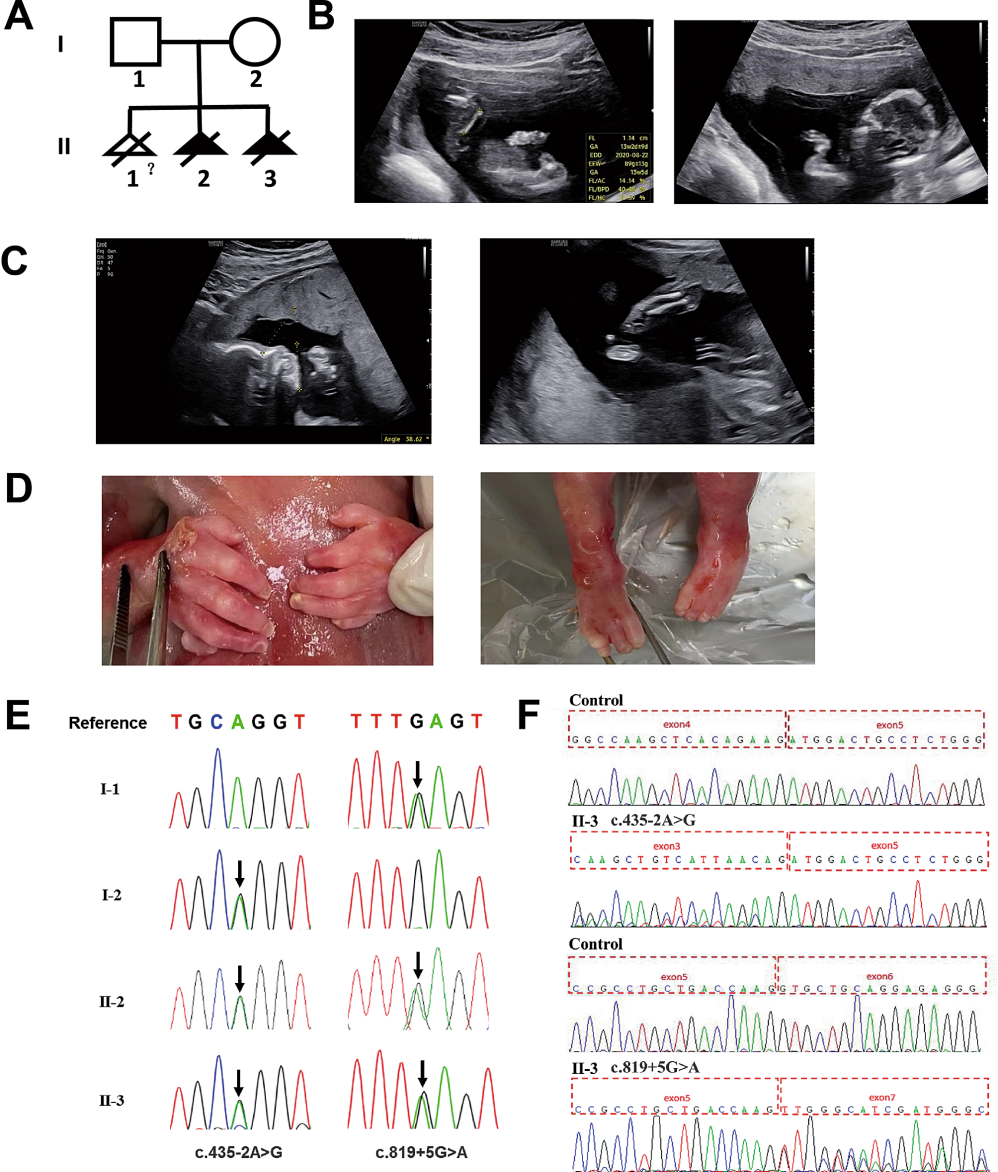
Clinical information and genetic analysis of case 8. **A**. The pedigree of the family. **B**. The ultrasound images of the fetus II-2. Left panel shows short femur (Femur length = 1.14 cm), and right panel shows short and in-curving forearm. **C**. The ultrasound images of the fetus II-3. Left panel shows micrognathia, and right panel shows short and bowing ulna and radius. **D**. The fetal autopsy images of the fetus II-3. Left panel shows incomplete development of fifth digits of both hands with tiny accessory bones present laterally, and right panel shows missing fifth digits of both feet. **E**. Sanger sequencing of *DHODH* in family members revealed variants in the fetuses and their parents. Variants were indicated by black arrows. **F**. RT-PCR and Sanger sequencing of cDNA from the fetus II-3 shows the c.435–2A> G variant altered the canonical splice acceptor site of intron 3, resulting in skipping of exon 4, and the c.819+5G>A variant altered the donor splice region of intron 6, leading to skipping of exon 6

The couple are healthy and non-consanguineous. The mother (“gravida 3, para 0”, G3P0) had three pregnancies, and she experienced an induced abortion of her first pregnancy (Fig. 4A). Her second pregnancy was terminated due to cystic hygroma, short and bowed forearms, and short femur detected by fetal ultrasound scan at 15 weeks of gestation (Fig. 4B). No genetic analysis was performed at that time. During her third pregnancy, routine mid-trimester fetal ultrasound scan at 23^+ 4^ weeks of gestation suggested micrognathia, short and bowed ulna and radius (Fig. 4C). The pregnancy was terminated at 24^+ 1^ weeks of gestation and autopsy was performed. In addition to the features observed by prenatal ultrasound scan, the fetus displayed hypoplasia of the fifth digits of both hands and bilateral absence of fifth digits of both feet (Fig. 4D).

Quad WES was performed with DNA from two fetuses and their parents, and two compound heterozygous splicing variants of *DHODH* gene were identified. Sanger sequencing confirmed the variants in both fetuses and the mother was heterozygous for the c.435–2A>G variant, and the father was heterozygous for the c.819+5G>A variant (Fig. 4E). These two variants were not recorded in the 1000 Genomes Project database, dbSNP, or gnomAD. Furthermore, RNA analysis was performed to analyze the splicing effects of these variants. PCR with reverse transcription (RT-PCR) of RNA from the fetus II-3 and the control in combination with Sanger sequencing revealed that the c.435–2A>G variant caused skipping of exon 4 in mRNA splicing and subsequent premature termination codon in exon 6, and the c.819+5G>A variant led to skipping of exon 6 and in-frame deletion of 38 amino acids, which removes < 10% of protein (Fig. 4F). According to the ACMG variant classification guideline [11], the c.435–2A>G variant could be classified as P with 1 very strong (PVS1) and 2 supporting (PM2_Supporting and PP1) points of evidences, and the c.819+5G>A variant could be classified as LP with 2 moderate (PVS1_Moderate, PM3) and 2 supporting (PM2_Supporting and PP1) points of evidences.

### Case 259

**Fig. 5:**
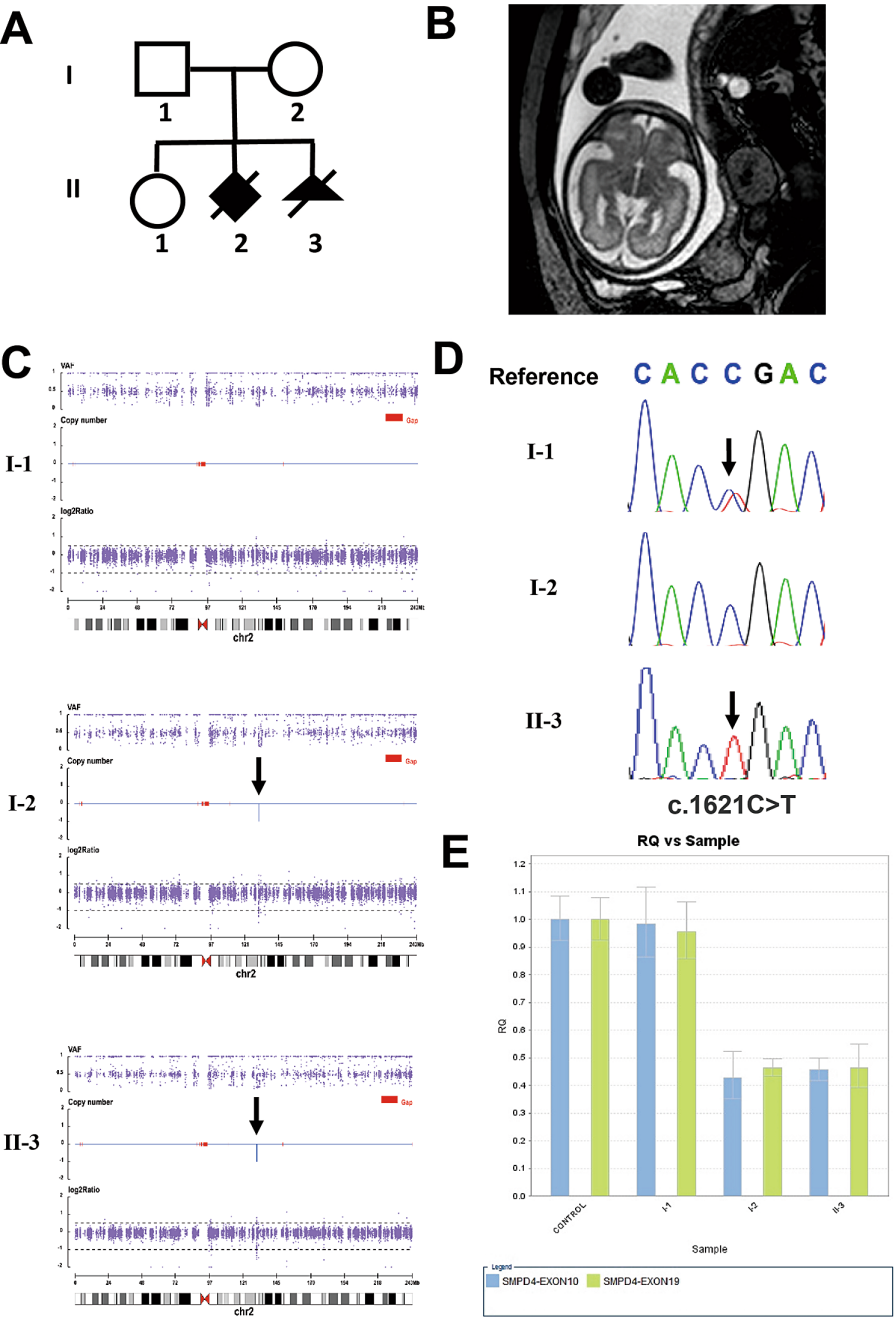
Clinical information and genetic analysis of case 259. **A**. The pedigree of the family. **B**. The MRI image of the fetus II-3 shows simplified gyration at 32^+ 3^ weeks of gestation. **C**. Exome-based CNV analysis identified the heterozygous microdeletion on chromosome 2 of the mother I-2 and the fetus II-3, as indicated by black arrows. **D**. Sanger sequencing of *SMPD4* in family members revealed the c.1621C>T variant in the fetus II-3 and the father I-1, as indicated by black arrows. **E**. Exon10 and exon19 of *SMPD4* gene were selected and amplified by qPCR to validate the 40Kb deletion on chromosome 2q21.1. The numbers in Y-axis indicated the 2-ΔΔCT value. Heterozygous deletion of *SMPD4* exon 10 and 19 were detected in the mother I-2 and the fetus II-3 compared with the control sample

The parents are healthy and non-consanguineous. The mother (“gravida 3, para 2”, G3P2) had three pregnancies. Her first child is a healthy girl. Her second child was delivered at 37 weeks of gestation by cesarean section due to a risk of uterine rupture. The newborn had seizures on the second day after birth and a magnetic resonance imaging (MRI) at 3 days after birth showed simplified gyral pattern. The newborn died shortly after birth (Fig. 5A). During her third pregnancy, fetal MRI scan at 25^+ 3^ weeks of gestation indicated relative small cerebellum, while anomaly of gyral pattern was not observed. Fetal MRI scan at 32^+ 3^ weeks of gestation suggested thin corpus callosum, and simplified gyral pattern (Fig. 5B). The parents opted to terminate the pregnancy at 32^+ 6^ weeks of gestation.

Trio WES was conducted with DNA from the fetus and their parents, and detected a nonsense variant (c.1621C>T, p.Arg512Ter) of *SMPD4* gene, which is inherited from the father. Furthermore, exome-based CNV calling identified a maternally inherited 40 kb heterozygous deletion on chromosome 2 (chr2q21.1:130900841-130941539del) that encompassed the whole *SMPD4* gene (Fig. 5C). Sanger sequencing confirmed that the fetus was hemizygous for the c.1621C>T variant and the father was heterozygous for the c.1621C>T variant (Fig. 5D). Moreover, qPCR was performed and validated the heterozygous deletion of exon 10 and 19 of *SMPD4* gene in the fetus and the mother (Fig. 5E). These two variants were not recorded in the gnomAD. According to the ACMG variant classification guideline [11], the c.1621C>T variant could be classified as P with 1 very strong (PVS1) and 2 supporting (PM2_Supporting and PP4) points of evidences, and the whole gene deletion of *SMPD4* could be classified as P with 1 very strong (PVS1), 1 moderate (PM3) and 1 supporting (PM2_Supporting) points of evidences.

## Discussion

In this study, we retrospectively analyzed the WES results of fetuses from 375 families with pregnancy loss or fetal structural anomalies, and the overall detection rate was 32.3% (121/375). As aneuploidy and CNVs could be detected by exome-based CNV analysis, only WES was performed in this study, which identified chromosomal aneuploidy and triploidy in 7.5% (28/375) of cases, CNVs in 5.1% (19/375) of cases, and SNVs/Indels in 19.7% (74/375) of cases. Among these, LP or P CNVs accounted for 4.8% (18/375) and LP or P SNVs/Indels accounted for 15.2% (57/375) (Table 1). The overall diagnostic yield is 27.5% (103/375), and an additional 18 cases (4.8%, 18/375) had a probable genetic diagnosis with VUS CNVs or VUS SNVs/Indels. Many studies have demonstrated the utility of WES in fetuses, and the diagnostic yield varies widely due to differences of inclusion criteria, fetal phenotype and study size. A previous meta-analysis revealed that when karyotype or CMA is normal, the incremental diagnostic yield of WES for prenatal diagnosis of 4350 fetuses with fetal structural anomalies was 31% (95% confidence interval (CI): 26-36%, *p* < 0.0001), and the diagnostic yield of pre-selected cases with likelihood of monogenic disorders was significantly higher than that of unselected cases (42% vs. 15%, *p* < 0.0001) [14]. Recently, a systematic review indicated that the incremental diagnostic yield by exome or genome sequencing after karyotyping/CMA is 33% (95%CI: 27-40%) for 2120 cases with congenital anomalies or perinatal death [15]. In the Prenatal Assessment of Genomes and Exomes (PAGE) study, prenatal WES was applied to analyze a cohort of 610 fetuses with structural anomalies after exclusion of aneuploidy and CNVs, which yielded a diagnostic yield of 8.5% (52/610) [16]. In our cohort, most of the samples were cases that had undergone pregnancy termination due to fetal structural anomalies, while many cases in the PAGE study were ongoing pregnancies. In this study, the detection rate of diagnostic SNVs/Indels (17.9%, 56/313) in the subgroup of terminated pregnancies is higher than 12.5% as reported by the PAGE study, which may be explained by the different severity of the fetal phenotypes. The detection rate in the subgroup of stillbirth was 6.9% (2/29), which is comparable to the detection rate of 8.5% (21/246) in cases of stillbirth as reported by a previous study [17]. And in the subgroup of miscarriage, the diagnostic yield of aneuploidy and triploidy was the highest (18.2%, 6/33) for chromosomal abnormalities may account for up to 60% of cases of early pregnancy loss [18]. As families that have another affected family member or have experienced recurrent pregnancies with similar phenotypes are more likely to choose quad WES, the detection rate of quad WES was the highest (55.6%, 5/9) compared with fetal-parental trio or fetus-only WES (Table 1).

The frequency of genetic diagnoses varied significantly between phenotypic subgroups. The highest frequency of aneuploidy and triploidy occurred in fetuses with increased NT (45.5%, 5/11), and the highest diagnostic rate of CNVs was identified in fetuses with multisystem anomalies (10%, 12/120) (Fig. 1). Fetuses with skeletal anomalies exhibited the highest yield of diagnostic SNVs/Indels (56.7%, 17/30), which is consistent with a previous meta-analysis that the highest diagnostic yield was identified in fetuses with skeletal abnormalities (53% [95% CI 42–63%], *p* < 0.0001) [14]. Therefore, the most prevalent variants were identified in genes related to skeletal dysplasia, including *FGFR3*, *COL1A2*, *COL1A1*, *ALPL*, *DYNC2H1* and *FGFR2* (Fig. 3).

Of SNVs/Indels identified in 74 families, the most common inheritance pattern was de novo (36/74, 48.6%) (Table 2, Supplementary Table 3), suggesting low recurrence risk in future pregnancies, despite the possibility of gonadal mosaicism or low-level parental mosaicism. The variants detected in the remaining 38 cases had been inherited from parents (Table 2, Supplementary Table 3), suggesting high risk of recurrence, and preimplantation genetic testing or invasive prenatal diagnosis were recommended for these families. In addition, mosaic variants were also identified in this cohort. For example, WES detected a heterozygous variant c.2T>A in *DCC* gene of the fetus in case 116, and the mother was mosaic (7%, 8/110) for this variant (Supplementary Table 3). And pyrosequencing confirmed and quantified the presence of the c.2T>A variant in approximately 7% of the mother’s peripheral blood and saliva respectively (data not shown).

Exome-based CNV analysis is one of the strengths of our study, which detected not only large CNVs, but also small deletions or duplications. Karyotype analysis or CMA may not be necessary, thus reducing the cost and turnaround time. But the boundaries of CNVs are not precise due to the design of WES capture probes, and small deletions or duplications should be confirmed by qPCR (Fig. 5E). Furthermore, RNA analysis was conducted to verify the effects of splicing variants on non-canonical splice sites (Fig. 4F).

However, this study also has some inherent limitations. Our cohort is a selected cohort of deceased fetuses skewing towards pregnancy termination due to fetal structural anomalies, and not all fetuses with structural anomalies were included. Moreover, compared with postnatal phenotypes, our knowledge of prenatal phenotypes is limited due to restrictions to the phenotypes that can be identified by ultrasonography in utero, which makes it more difficult to obtain a genetic diagnosis. It is noteworthy that additional genetic evidence and follow-up functional investigations are needed for the interpretation of VUS variants, and reclassification of VUS variants is necessary as new evidence may continue to emerge in the future.

## Conclusions

In summary, the overall detection rate by WES in our cohort of fetuses with pregnancy loss and fetal structural anomalies was 32.3% (121/375), and the diagnostic yield is 27.5% (103/375). This study expands our knowledge of fetal phenotypes and mutation spectrum of various disorders, and aids the genetic counselling for future pregnancies. As our knowledge of human genome continues to grow and the cost of sequencing continues to decline, we recommend implementing WES into clinical practice for pregnancy loss and fetal structural anomalies.

## Electronic supplementary material

Below is the link to the electronic supplementary material.

**Supplementary table 1.** The characteristics of cases with aneuploidy or triploidy

**Supplementary table 2.** The characteristics of cases with CNVs

**Supplementary table 3.** The characteristics of cases with SNVs/Indels

**Supplementary table 4.** The characteristics of unresolved cases

## Data Availability

All datasets generated for this study are included in the article. The genotyping data for this article are not publicly available to assure patient confidentiality and participant privacy. Requests to access the datasets should be directed to TW, biowt@njmu.edu.cn.

## Abbreviations

WES: Whole exome sequencing
CNV: Copy number variation
LP: Likely pathogenic
P: Pathogenic
VUS: Variant of uncertain significance
SNV: Single-nucleotide variants
Indel: Insertion or deletion
CMA: Chromosomal microarray analysis
qPCR: Quantitative PCR
gnomAD: Genome aggregation database
OMIM: Online mendelian inheritance in man
HGMD: Human gene mutation database
ACMG: American college of medical genetics and genomics
ClinGen: Clinical genome resource
PCR: Polymerase chain reaction
RT-PCR: PCR with reverse transcription
cDNA: Complementary DNA
NT: Nuchal translucency
MRI: Magnetic resonance imaging
CI: Confidence interval
PAGE: Prenatal assessment of genomes and exomes
